# A case of multiple myeloma with markedly straight lines or curved deep groove nuclei

**DOI:** 10.1002/jha2.362

**Published:** 2021-12-05

**Authors:** Chang He, Ling Zhang, Zailin Yang

**Affiliations:** ^1^ Department of Laboratory Medicine Sichuan Chengdu Chengfei Hospital Sichuan China; ^2^ Key Laboratory of Laboratory Medical Diagnostics Designed by the Ministry of Education School of Laboratory Medicine Chongqing Medical University Chongqing China; ^3^ Center of Haematological Malignancy Chongqing University Cancer Hospital Chongqing China


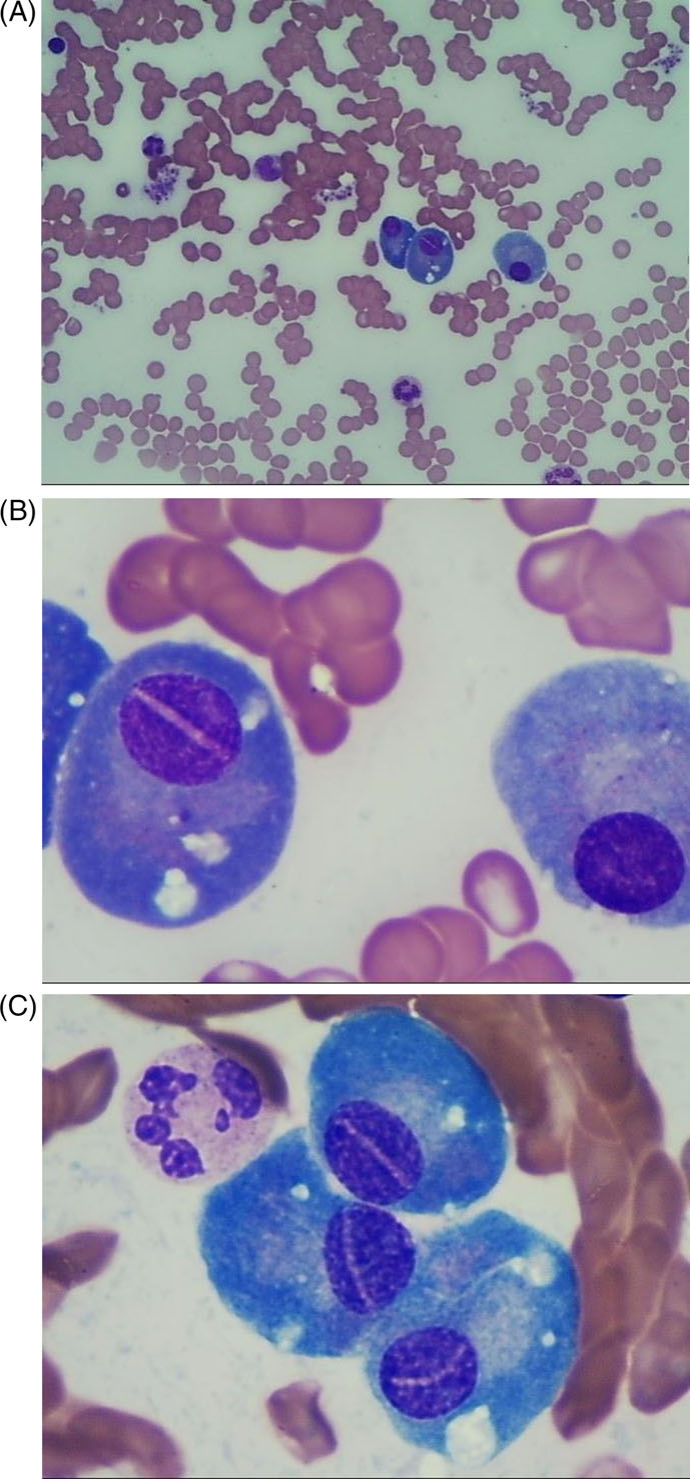


A 62‐year‐old male presented to the hospital with spontaneous intracerebral hemorrhage. A full blood count showed a total leucocyte count of 5.54 × 10^9^/L, hemoglobin concentration of 117 g/L, and platelet count of 147 × 10^9^/L. In bone marrow aspirate films, the proliferating plasma cells were predominantly observed, accounting for 61.5% of the total nucleated cells. Strikingly, straight lines or curved deep nuclear grooves were markedly noted in 28% of plasma cells (Figure 1, panels A–C; original magnification 1000×; Wright–Giemsa stain). Laboratory evaluation revealed monoclonal M protein with the elevated IgA of 43.8 g/L. Abnormal monoclonal bands in IgA and κ lanes were found in immunofixation electrophoresis. Flow cytometry identified 1.54% of κ‐restricted plasma cells expressing CD38, CD138, and CD56, which was also showed in bone marrow biopsy by immunohistochemistry. The skeletal examination by CT suggested multiple osteolytic bone destruction. Cytogenetic test showed a normal 46, XY karyotype. A diagnosis of multiple myeloma was made.

In summary, we describe a new case of multiple myeloma, characterized by the presence of numerous plasma cells with markedly straight lines or curved deep nuclear grooves in bone marrow.

Figure 1 (A) 400× Objective, Wright–Giemsa stain. (B and C) 1000× objective, Wright–Giemsa stain

